# A Sintering–Resting Strategy of Microwave Heating for Lithium Hydride Ceramic Based on Numerical Analysis of Thermal Effects

**DOI:** 10.3390/ma18122832

**Published:** 2025-06-16

**Authors:** Wenyan Zhang, Huayan Chen, Maobing Shuai, Xiangguo Zeng, Bin Huang

**Affiliations:** 1 College of Architecture and Environment, Sichuan University, Chengdu 610065, China; 2 Key Laboratory of Deep Underground Science and Engineering, Ministry of Education, Sichuan University, Chengdu 610065, China; 3 Institute of Materials, China Academy of Engineering Physics, Mianyang 621907, China

**Keywords:** lithium hydride ceramics, microwave sintering, thermal stress, electromagnetic–heat-force coupling

## Abstract

Lithium hydride (LiH) is one promising material for nuclear reactor shielding due to its high hydrogen content, but its poor mechanical strength and thermal conductivity pose challenges for fabricating large, crack-free ceramic components via conventional sintering. This study explores microwave sintering as a potential solution to enhance heating uniformity and reduce thermal stress during densification of bulk LiH ceramics. Using implicit function and level set methods, we numerically simulated the microwave field distribution and thermal response in both stationary and rotating samples. The results show that rotational heating improves temperature uniformity by up to 12.9% for specific samples, although uniform temperature control remains difficult through rotation alone. To mitigate stress accumulation from thermal gradients, we propose a cyclic sintering–resting strategy, which leverages LiH’s tensile strength–temperature envelope to guide safe and efficient processing. This strategy successfully reduced total sintering time from several days to 1.63 h without inducing cracks. Our findings offer practical insights into optimizing microwave sintering parameters for large-scale LiH ceramic production and contribute to enabling its application in advanced nuclear shielding systems.

## 1. Introduction

LiH ceramics, which are currently one of the most important neutron shielding materials, have attracted considerable research interest, owing to their high hydrogen storage capacity (up to 12.6 wt%) [1,2,3]. Despite their increasing importance in hydrogen energy, large-size or thick-walled LiH products cannot be easily sintered through traditional means. The reason could be mainly summarized with three points. First, the poor thermal conductivity will induce large temperature gradient. Moreover, LiH exhibits a high coefficient of thermal expansion, which could contribute to significant temperature stress, even with a small temperature gradient [4,5,6,7,8,9]. Finally, the low strength of LiH, especially at high temperature, can lead to cracking during the cooling, and even the heating, process [5,10,11,12]. Therefore, significant efforts are still required to improve both the safety and sintering efficiency of thick-walled, large-sized bulk LiH. Compared to traditional heating methods, microwave heating, which was first proposed in1960s [13] has the advantages of uniform heating and fast heating rate [14,15,16,17]. External microwave excitation is a promising method for sintering LiH, expected to produce rapid and uniform heating.

Scientifically, since the cause of cracking is the thermal stress generated by the temperature gradient, and the intensification of the temperature gradient is caused by the imbalance between microwave heating and heat conduction mechanisms, the competitive relationship between these two mechanisms of LiH is very crucial; however, they cannot be separated or obtained in an experimental way. Technically, the microwave sintering of LiH necessarily requires a suitable sintering strategy and the corresponding process parameters through the whole period.

LiH shares some common characteristics of dielectric materials, which have been demonstrated to be heated by microwave heating under the excitation of a microwave field [18]. Its rapid microwave densification sintering shows promising application prospects in the preparation of large-sized, thick-walled LiH products [19,20]. Studies have explored the mechanical and thermodynamic mechanisms of LiH through microscopic first-principles approaches. For instance, Gao et al. [21] investigated the stability and electronic structure of LiH and predicted hydrogen diffusion behavior, while Yu and Pan [22] modified the stability and structural properties of LiH via doping. Relatedly, studies have focused on the mechanical properties of bulk LiH as a structural material. Shi et al. [23]. observed that LiH ceramics exhibit brittle fracture at low temperatures, while demonstrating creep fracture and ductile fracture at elevated temperatures. Additionally, Peter W.F. Evans [24] successfully fabricated LiH in various sizes and shapes using pressureless sintering, with a relatively long production cycle. Meanwhile, Lu et al. [19,20] achieved preliminary success in sintering LiH samples through microwave sintering, though their research primarily focused on the construction of industrial microwave furnaces. While these studies provide valuable early insights into the LiH sintering, their applications are largely constrained in specific industrial settings or require long production cycles. More recently, Yan et al. [25] investigated the cooling process in microwave sintering of LiH and significantly reduced the cooling cycle of LiH using the PFAD method, while ensuring safety requirements. However, the heating process in microwave sintering of LiH still lacks detailed and in-depth quantitative analysis from a theoretical perspective.

This study aims to explore the electromagnetic–heat-force mechanism in the microwave sintering process, find out the process parameters that meet the strength conditions, and on this basis, shorten the sintering time, optimize the sintering efficiency, and provide a theoretical basis and process parameter guidance for practical application. Our study, for the first time, ingeniously decoupled and quantified the two mechanisms leading to the increasing temperature gradient of LiH by the numerical method, then proposed a novel sintering–resting strategy of microwave heating and examined whether it can help resolve sintering fractures and inefficiencies. These achievements will provide deeper insights into a practical and effective solution for sintering large-block irregular-shaped LiH ceramics in engineering.

This paper first analyzes the key factors determining microwave field distribution and heating effects from the governing equations of the cavity’s electromagnetic field, clarified in Section 2. Through numerical simulations of a simplified cavity and sintered structure geometry, standing electromagnetic wave fields were obtained, validating the model’s rationality, shown in Section 3. Section 4 lists and discusses the main results of this paper, including electromagnetic fields (Section 4.1), the temperature fields, and its uniformity, which was discussed through the COV method and improved by rotary heating (Section 4.2), and the sintering–resting strategy proposed based on stress intensity conditions, as the most important achievement of this work (Section 4.3). In detail, we model the competing relationship among those key factors in the response mechanisms to design a quantitative system in Section 4.3.1, which allows us to precisely control the heating and resting temperature and time lag. Based on comprehensive quantification of microwave heating and thermal conduction, a cyclic heating strategy termed “sintering–resting” is proposed in Section 4.3.2, and a design scheme of heating parameters is given in Section 4.3.3.

## 2. The Basic Equations of Electromagnetic–Thermal-Force Coupling in Microwave Sintering

### 2.1. The Basic Equations for Microwave Electromagnetic Field

To understand the heating behavior of materials subjected to microwaves, it was first necessary to examine the electromagnetic field within the microwave cavity.

The electromagnetic field in a microwave cavity can be mathematically described by the functions of electric field intensity *E* and magnetic field intensity *B*, varying with position *X* and time t, and denoted as *E* (*X*, *t*) and *B* (*X*, *t*). Microwave electromagnetic waves with a specific frequency and power pass through a metal shell channel and enter the interior of the metal cavity. Due to the shielding effect of the cavity wall, the microwaves will be reflected once they reach the cavity wall, forming an uneven electromagnetic field.

The electromagnetic field, described as *E* (*X*, *t*) and *B* (*X*, *t*), serves as the solution of the Helmholtz equation as the governing equation, and can be defined by the total reflection boundary conditions, which serves as a boundary value problem for the wave equation in three-dimensional conditions. Finite element software was employed to obtain a numerical solution of these equations by discretizing time and space.

The governing equations of an electromagnetic field can be described by Maxwell’s equations:

(1) $$\nabla \times \vec{H}=\vec{J}+\varepsilon \frac{\partial \vec{E}}{\partial t}$$

(2) $$\nabla \times \vec{E}=-\mu \frac{\partial \vec{H}}{\partial t}$$

(3) $$\nabla \cdot \vec{B}=0$$

(4) $$\nabla \cdot \vec{D}=\rho_{c}$$

(5) J=σE

where $\vec{E}$ represents the electric field intensity, $\vec{H}$ represents the magnetic field intensity, ε represents the permittivity of LiH, μ is the permeability, $\vec{D}$ represents the electric displacement vector, $\vec{B}$ is the magnetic induction intensity, $\vec{J}$ is the current density, $\rho_{c}$ represents the charge density, t represents time, and σ denotes the electrical conductivity.

Equation (1), termed the Ampere law, reveals the relationship between the electromagnetic field, dielectric constant, and current density. As shown in Equation (5), the current density was directly proportional to the conductivity. Equation (2), known as the Faraday law of magnetic induction, illustrates the relationship between the electromagnetic field and permeability. The coupling of permeability, conductivity, and dielectric properties with the external electromagnetic field formed an electromagnetic field in space and in the medium, and these three factors were affected by various other properties.

For the setting of the boundary conditions of the electromagnetic field, except for the waveguide feed port, the entire cavity surface is regarded as an ideal electrical conductor (PEC), and the boundary conditions are assumed as follows:

(6) $$\vec{n}\times \vec{E}=0$$

where $\vec{n}$ is the unit normal.

### 2.2. The Basic Equation of Microwave Heating and Thermal Transfer

Theoretically, the dielectric properties were found to be important to the heating effect of materials during microwave heating, and were crucial to the distribution of the microwave field. The expression of dielectric properties is given by Equation (7):

(7) $$\varepsilon^{*}=\varepsilon^{\prime }-j\varepsilon^{\prime \prime }$$

where $j=\sqrt{-1}$, and $\varepsilon^{\prime }$, $\varepsilon^{\prime \prime }$ denote the dielectric constant and dielectric loss factors, respectively.

At each point of the substance, taking into account the unit volume, the intensity of the electric field acted on the dielectric material, resulting in electromagnetic losses. These losses of power, which is the general concept of power density, can be obtained in the following ways:

(8) $$W=\frac{1}{2}\omega \varepsilon^{\prime \prime }E_{0}^{2}$$

where $\varepsilon^{\prime \prime }$ represents the imaginary part of the complex permittivity of the dielectric material. The energy lost by the electromagnetic microwave was converted into the heat consumed by the dielectric material, which increased the temperature of the material.

Then, the increase in temperature at the heated point was calculated using the following heat conduction equation:

(9) $$\frac{\partial T}{\partial t}=\frac{W}{\rho c_{p}}+\frac{k}{\rho c_{p}}\nabla^{2}T$$

where $c_{p}$ is the heat capacity of the material, T is the temperature of the material, k is the thermal conductivity, and ρ denotes the density. Consequently, this increase in temperature included not only the heating effect determined by the power loss described in the first term on the right, but also the heat conduction determined by the temperature gradient described in the second term on the right.

During the microwave heating process, each material point can be regarded as a heat source in the temperature field, as shown below.

(10) Q=Wdtdv

In addition, on the surface of the block, the heat in LiH block ceramics can also be transferred to the environment through thermal convection and thermal radiation. In the simulation, the heat convection and the thermal radiation are considered as the boundary conditions, which are given below.

Heat convection occurs at the interface of the ceramic and the shielding gas, and the heat flux q can be described by Newton’s law of cooling:

(11) $$q=-\alpha (T_{w}-T_{a})$$

where $T_{a}$ is the temperature of the fluid, i.e., the temperature of the argon, and $T_{w}$ is the ceramic temperature of the surface, which changes continuously during the heat transfer process.

Heat radiation is described by the Stefan–Boltzmann law:

(12) $$q=-\eta \varphi (T^{4}-T_{f}^{4})$$

where η is the Stefan–Boltzmann constant (5.67 × 10^−^⁸ W/m^2^·K^4^), φ is the coefficient of emissivity, and $T_{f}$ is the temperature of the furnace. The emissivity coefficient, while behaving as a material property, is actually dependent on multiple factors, including the wavelength of emitted radiation, material temperature, and emission angle. This dimensionless parameter ranges from 0 to 1. For ceramic materials, as evidenced by previous studies [26,27], typical emissivity values range between 0.6 and 0.9. To reduce computational complexity in our model, we adopted a fixed emissivity value of 0.7 as a reasonable approximation.

### 2.3. The Basic Equations for Thermal Stresses

Different amounts of expansion were generated with different amounts of temperature increase. The unevenness in thermal expansion resulted in internal stresses, generally expressed by

(13) σ=α⋅(ΔT)⋅E

where α denotes the coefficient of thermal expansion, E is the Young’s Modulus, and σ denotes the stress, which critically differs from previous definitions. These internal stresses are known as temperature stresses. Stress greater than strength can cause a material to crack. Unfortunately, LiH ceramics have significantly poor strength; therefore, the temperature and stress field should be carefully monitored and controlled in the microwave heating process.

**Figure 1:**
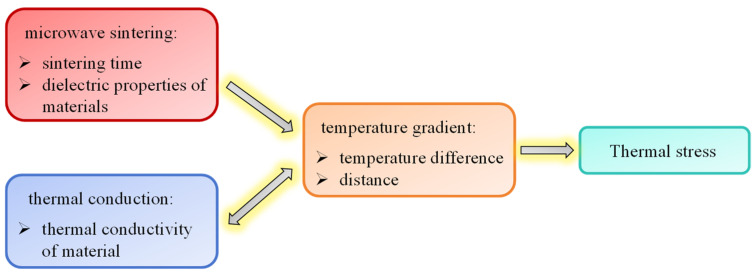
Coupling relationship between the thermal effect and sintering stress in the microwave sintering process.

Generally, the coupling relationship between the thermal effects and sintering stress during microwave sintering is illustrated in Figure 1, and this served as the basis of this study. The thermal stress was determined by the temperature gradient. The temperature-dependent dielectric properties of the material allowed it to be heated per unit time. Furthermore, as the sintering time extends, heat is also constantly accumulating. Therefore, the temperature gradient intensifies. In contrast, the thermal conductivity driven by the temperature gradient will relieve itself. Consequently, the transient temperature response of substances under microwave action essentially consisted of a coupled response process involving both electromagnetic waves and heat transfer, as illustrated in Figure 1.

## 3. Numerical Modeling and the Simulation Method

### 3.1. The Setup of the Microwave Oven Model

**Figure 2:**
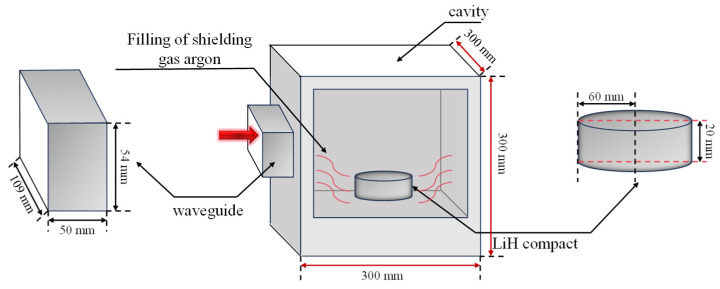
Diagram of the microwave oven and LiH sintered body.

A schematic diagram of the numerical model for the microwave sintering of LiH ceramics is shown in Figure 2. Figure 2 shows the simulated microwave sintering system, which mainly consists of four parts: the microwave-sintering furnace chamber, the waveguide, the protective gas, and the LiH sample. The temperature of LiH during the heating stage was increased and monitored by using a microwave-sintering furnace chamber. The rectangular waveguide port had dimensions of 109 mm × 54 mm and a length of 50 mm. Along the length direction, TE10 mode microwaves with a frequency of 2.45 GHz were fed, with a power of 1000 W. In the cavity, LiH compacts were placed on the bottom, measuring Φ60×20 in size.

This study utilizes the coupling of the electromagnetic wave module, solid heat transfer module, and solid mechanics module in COMSOL Multiphysics 6.1 software to simulate the microwave heating process and to solve it using a frequency-domain transient solver. The CPU model is Intel (R) Xeon (R) Platinum 8370C CPU @ 2.80 GHZ, and the memory is 256 GB. The distributed heat source is calculated in the steady-state frequency-domain electromagnetic analysis, followed by transient heat-transfer simulation and transient structural-mechanics simulation to demonstrate how heat and thermal stress redistribute in LiH. The element size is determined by the Nyquist criterion:

(14) $$S_{\max}=\frac{\lambda_{\min}}{2}$$

In the formula, $\lambda_{\min}$ is the minimum wavelength (mm), and $S_{\max}$ is the maximum element size. According to this criterion, in this study, the maximum element size was set at 6 mm, and the mesh quality of LiH was refined. The minimum element quality obtained was 0.2151, which was greater than 0.1, and the mesh quality met the requirements. The convergence criterion is specifically set as follows: the residuals of all equations are less than the tolerance of 1 × 10^−6^, and the maximum number of iterations is 50.

The simulation was performed by a frequency-transient solver with the maximum time step set to 0.25 s, and the initial temperature in the microwave cavity set to 20 °C. The convective boundary was assumed for the upper surface of the LiH sample, with the heat transfer coefficient set to 10 $W/(m^{2}\cdot K)$ [28] Due to the short heating time, other surfaces were set as thermally insulated.

**Figure 3:**
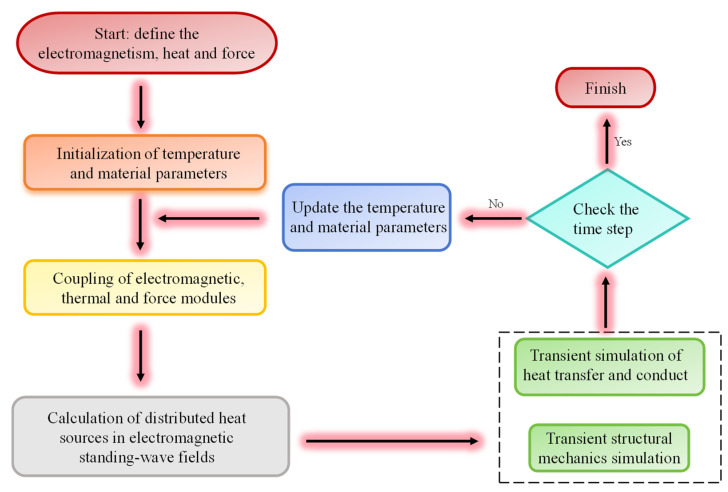
The step diagram of COMSOL simulating microwave heating of LiH.

Figure 3 shows the steps of simulating microwave heating of LiH using COMSOL.

### 3.2. Material Properties Varied with Temperature

Assuming that the initial temperature of the LiH sintered sample was 20 °C and it was thermally insulated, we sintered the specimen from room temperature (20 °C) to 520 °C through microwave heating [6]. In the simulation, as the material is considered to be constructed from randomly arranged powder, isotropic assumption is chosen. Meanwhile, noting its high brittleness, LiH is assumed to be elastic. Table 1 lists the material properties used in the simulation, indicating that most properties were closely related to the temperature. Therefore, using temperature as the independent variable and various physical properties as the dependent variables, interpolation functions were constructed using the cubic spline interpolation method. The dielectric properties in both the waveguide and the cavity were set as a vacuum, where the relative permittivity and permeability were both taken as 1, and the electrical conductivity was taken as 0 S/m.

Within the temperature range of room temperature up to 700 °C, the dielectric permittivity, dielectric loss, and dielectric loss tangent of LiH increase with temperature [30]. The dielectric permittivity of LiH powders with different particle sizes exhibits complexity. Specifically, LiH powders in the particle size range of 0.6–0.9 mm demonstrate favorable wave-absorbing properties. The differences in dielectric properties among the powders of various sizes are not obvious; thus, we utilize the fitted results [30] as working parameters for simulating the microwave heating of LiH.

### 3.3. The Implicit Method in Modeling a Rotary Sample in Microwave Heating

Rotary heating is a common means of improving the uniformity of microwave heating. For simulation, if the rotational motion is defined, the grid consists of a discrete physical domain that must be constantly updated, requiring significant computation, and mesh mismatch. The implicit function and level set method proposed by Ye et al. [31] were employed to carry out the simulation for a rotary sample. Simple, but not without subtleties, this method could significantly improve the computational efficiency and accuracy. The physical domain can be divided into the exterior domain, interior domain, and boundary, through the Heaviside function, as follows:

(15) $$H(\phi )=\left\{\begin{array}{c} 0,\phi \leq 0\\ 1,\phi >0 \end{array}\right.$$

where ϕ depends on the coordinates of points. The values of the Heaviside function equal to 0 and less than 0 denoted the boundary and interior domain, respectively, while the values of the Heaviside function greater than 0 denoted the exterior domain.

The coordinates of any given point in the sample under rotational motion could be expressed as

(16) $$\left\{\begin{array}{c} x_{1}=(x_{0}-x_{c})\cdot \cos\theta +(y_{0}-y_{c})\cdot \sin\theta +x_{c}=x_{0}\cdot \cos(\omega_{p}t)+y_{0}\cdot (\sin\omega_{p}t)\\ y_{1}=-(x_{0}-x_{c})\cdot \sin\theta +(y_{0}-y_{c})\cdot \cos\theta +y_{c}=x_{0}\cdot \cos(\omega_{p}t)+y_{0}\cdot (\sin\omega_{p}t)\\ z_{1}=z_{0} \end{array}\right.$$

where $\omega_{p}$ is the angular velocity of rotation, $\left(x_{0},y_{0},z_{0}\right)$ represents the initial position of that point, $\left(x_{1},y_{1},z_{1}\right)$ is the coordinate after a unit time step, and $\left(x_{c},y_{c})=(0,0\right)$ is the center of rotation.

The equivalent dielectric constant for the above rotational motion could be expressed by

(17) $$\varepsilon_{r}=\varepsilon_{p}\cdot (1-H(\phi ))+\varepsilon_{a}\cdot H(\phi )$$

where $\varepsilon_{p}$ and $\varepsilon_{a}$ are the relative permittivity of LiH and air, respectively. The distribution of heat sources could be obtained by calculating the power dissipation of the microwaves in the LiH sample.

## 4. Results and Discussion

### 4.1. The Electromagnetic Field in the Microwave Cavity

**Figure 4:**
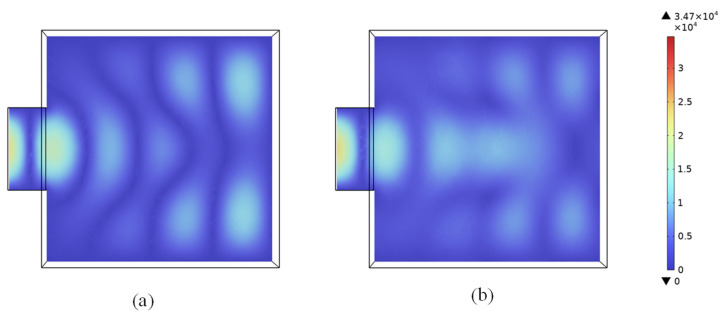
Electric field model E (V/m) cloud map: (**a**) without LiH compact, (**b**) with LiH compact.

Figure 4 shows the distribution of the electric field model inside the microwave oven before and after placing the LiH compact. We observed that the electric field distribution was non-uniform, and the results also confirmed the existence of peak and valley spots (termed hot and cold spots) of the microwave field. When the LiH compact sample was placed in the microwave oven, the electromagnetic field distribution inside the cavity theoretically changed, due to the magnetic, electrical, and dielectric properties of the dielectric material. However, because the relative magnetic permeability of LiH was still set as 1, the electrical conductivity was very low (0.03 S/m) and the dielectric loss factor was small (0.01–0.04), with a relatively small volume of the LiH compact, where the placement of the dielectric had little influence on the electromagnetic field inside the cavity, as indicated in Figure 4b.

As the sample was heated by the standing microwave, a spot at the peak of the microwave field was consistently subjected to intense heating, while the spot at the valley of the microwave field was only slightly heated.

### 4.2. Microwave Heating Effect of LiH Ceramics

For the microwave heating effect, this section first discusses the temperature increase under the electromagnetic microwave. As the material property of LiH varies a lot, with not only temperature, but also the states of compact, for simplification, the heating effects are carefully checked with different dielectric parameters. Moreover, the thermal uniformity is discussed both by the temperature difference between the selected hot and cold points, and the temperature coefficient of variation (COV). Finally, the rotating samples were modeled to verify whether the method was sufficient to improve the temperature uniformity of LiH ceramics.

#### 4.2.1. The Temperature Distribution of the LiH Compact

**Figure 5:**
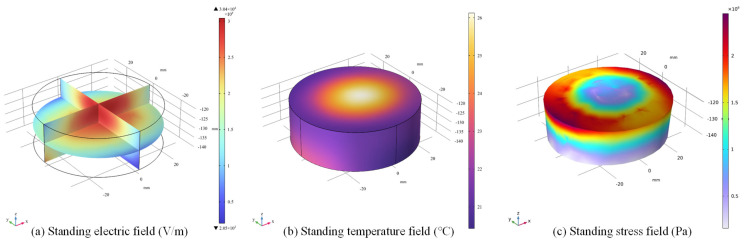
Standing electric field, temperature and stress of the LiH compact after 10 s of sintering.

To study the heating effect, the simulation is conducted with sintering time set to be 120 s and the dielectric loss factor set to be 0.01. The electric field intensity distribution, temperature distribution and stress distribution of LiH ceramics are illustrated in Figure 5, in which (a) is the standing electric field, and (b) is the temperature distribution, showing a characteristic decrease radially from the center, in consistency. From the stress field in Figure 5c, it can be found that the upper edge of the sample has the greatest thermal stress during the sintering process and is the part most prone to damage or even fracture.

**Figure 6:**
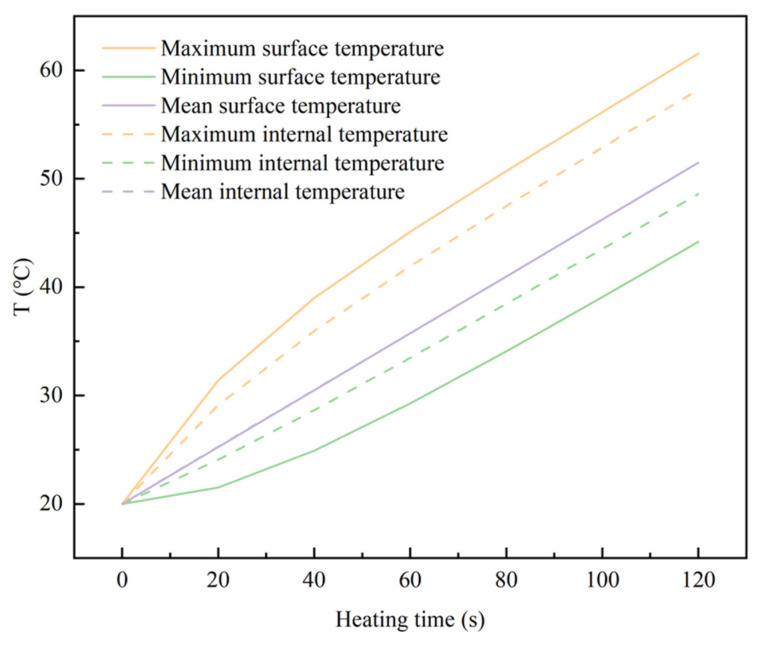
Temperature increase of the LiH sample under microwave heating.

Figure 6 plots the temperature history of the sample, indicating that the internal and external mean temperature both increase almost linearly, and the upward trends are almost exactly the same. From the beginning to the end, in 120 s, the maximum temperature at the hottest spot rose by approximately 40 °C, indicating it is indeed an effective heating method.

In more detail, the average heating rate is approximately 0.262 °C/s, the surface hot spot is approximately 0.301 °C/s, the internal hot spot is approximately 0.291 °C/s, and the heating rate at the cold spot on the surface and on the inside was 0.227 °C/s and 0.245 °C/s, respectively. It should be noted that it is effective heating, but not strictly uniform heating. Therefore, the level of the uniformity of temperature needs to be evaluated quantitatively, especially for the LiH ceramics, which could be damaged by relative small thermal stress.

Here, the nominal temperature gradient $\bar{\nabla T}$ and the COV are employed as follows:

(18) $$\bar{\nabla T}=\frac{T_{hot}-T_{cold}}{d}$$

where d is the distance between the hot and cold spots, which in this case was 37.498 mm. COV can be defined by

(19) $$COV=\frac{\sqrt{\frac{1}{N}\sum_{j=1}^{N}{\left(T_{j}-\bar{T}\right)}^{2}}}{\bar{T}-T_{0}}$$

where N is the total number of grid points in the LiH domain, $T_{j}$ is the temperature of the $j^{th}$ grid point of the sample, $\bar{T}$ is the average volume temperature of LiH, and $T_{0}$ is the initial temperature. Temperature coefficient of variation (COV) is a normalized measure of the dispersion of the temperature distribution, defined as the ratio of the standard deviation of each temperature measurement point to the mean. The smaller the COV value, the more uniform the temperature of LiH during heating.

#### 4.2.2. The Effect of Dielectric Property on Heating

Recalling Equation (7), the heating power depends on $\varepsilon^{\prime \prime }$ and $E_{0}$. The heating effect was determined by the electric field intensity as the load, and the dielectric parameters were determined by the material properties. In the standing- wave field, the field intensity distribution remains unchanged over time, and is constant. In other words, if the electric field at a certain point is high, then its heating power will also be high. This implies that points with high electric intensity would be heated more severely.

Although the literature [19] reports that the parameters change significantly with the increase in temperature (0.01 at 20 °C and 0.04 at 520 °C), due to the complex and variable properties of lithium hydride ceramics, it is difficult to predict the accurate values of the parameters when the external conditions are different. Therefore, the influence of different dielectric loss factor values (0.01–0.04) on the microwave heating effect was specifically investigated here. The simulation results with different dielectric loss factors including cold and hot point and its related nominal temperature gradient and COV are listed in Table 2.

It can be known from Table 2 that when the dielectric loss factor is increased 4 times, the temperature, even at the hot spot, has only increased 1.2 times, gradually from 26.055 °C to 31.478 °C, not to mention the temperature at the cold spot, with only a 2% increment. It means that the possible improving of the dielectric loss factor makes for limited improvement in microwave heating for LiH. Considering the uniformity of temperature, the nominal temperature gradient of LiH increased from 0.149 °C/mm to 0.284 °C/mm, with an increase of 90.6%, the COV increased from 0.35 to 0.43, and the uniformity decreased by 22.9%. Given the poor performance of LiH, this intensification of non-uniformity could be fatal to it.

#### 4.2.3. Microwave Heating at Different Positions and Rotary Heating

**Figure 7:**
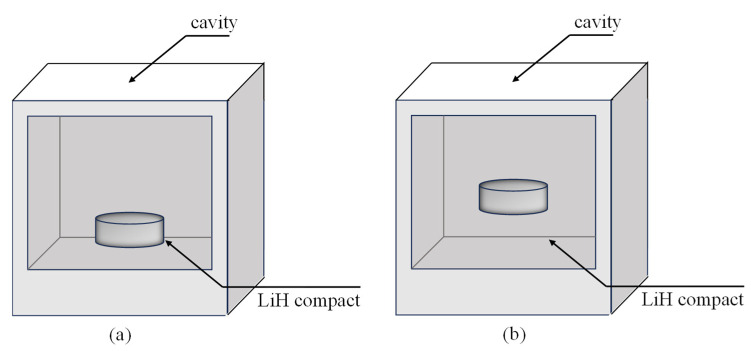
Placement of LiH during microwave rotational heating: (**a**) the sample was placed at the bottom of the microwave cavity, and (**b**) the sample was placed in the middle of the microwave cavity.

**Figure 8:**
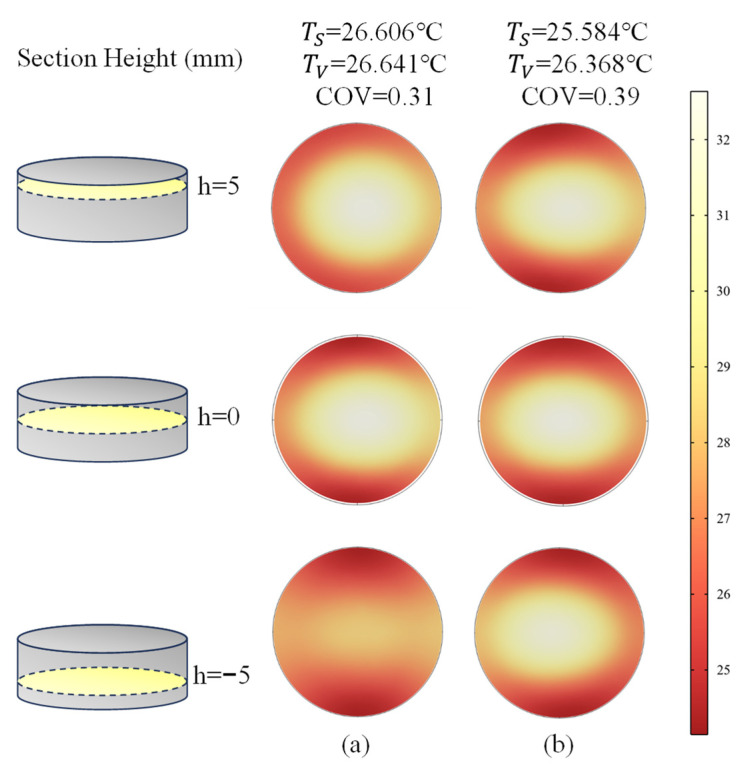
Temperature slice graph at heights of −5 mm, 0 mm and 5 mm for LiH samples after microwave heating for 20 s: (**a**) placed at the bottom and (**b**) placed in the middle.

We have tried various methods to smooth these temperature gradients. The LiH compacts were placed at different positions in the microwave cavity, as shown in Figure 7. Then, the simulated results, the temperature slice graph of LiH samples, is shown in Figure 8, where $T_{S}$ represents the surface mean temperature while $T_{V}$ denotes the volume mean temperature. The temperature slice graph shows heights of −5 mm, 0 mm and 5 mm for the LiH samples after microwave heating for 20 s, with (a) placed at the bottom and (b) placed at the middle, which may leave an impression on an uneven temperature field. More accurately, the COV is also calculated and listed in Figure 8. It shows 0.31 and 0.39 placed at the bottom and in the middle of the cavity, respectively, indicating that the bottom with a smaller COV value is in a better position, in this case.

**Figure 9:**
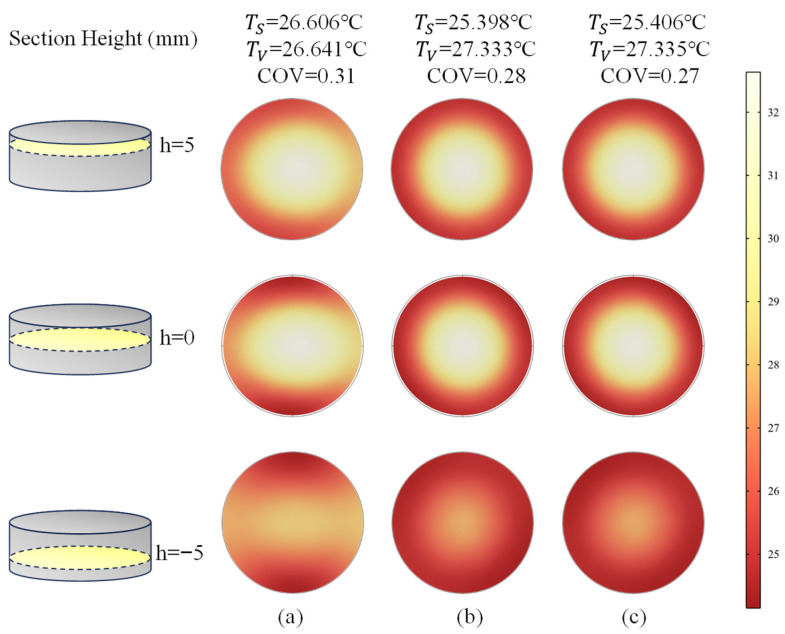
Temperature slice graphs at heights of −5 mm, 0 mm and 5 mm for LiH samples after rotary heating for 20 s with rotational speed of (**a**) ω = 0 rad/s, (**b**) ω = *π*/12 rad/s, (**c**) ω = *π*/6 rad/s.

**Figure 10:**
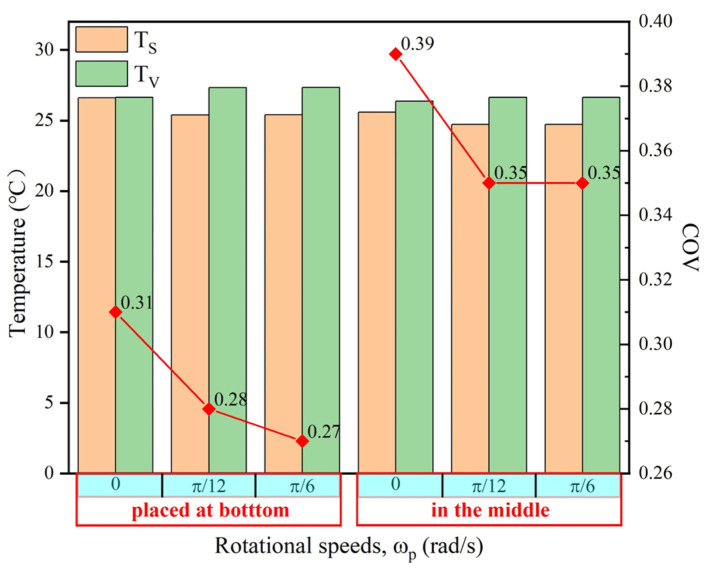
The temperature uniformity after 20 s of LiH microwave sintering.

The rotary heating placed at the bottom of the cavity were simulated with rational speeds of 0 rad/s π/12 rad/s, and π/6 rad/s, based on the implicit function and level set method. The corresponding results are shown in Figure 9. As with the centrosymmetric motion, the temperature distribution of the LiH sample was also centrosymmetric. Moreover, the rotational motion changed the temperature distribution of LiH markedly and reduced the COV effectively by 12.9%, when comparing Figure 9b with Figure 9a. The results also indicated that the rotational motion could improve the heating uniformity. This was consistent with the results obtained in the literature [32]. With the speeding up of the rotating, shown in Figure 9c, we observed that both the $T_{V}$ and COV have changed little, within 1%. The comprehensive results are plotted in Figure 10, where the bars show the mean temperature, and the curve shows the COV.

### 4.3. A Sintering–Resting Strategy of Microwave Heating Based on PFAD

It is easy to understand that the non-uniform temperature field will lead to harmful thermal stresses, but the critical stresses should be determined by the strength condition. Considering the tensile strength–temperature envelope of LiH, a process-based failure assessment diagram (PFAD) [25] proposed before by the researchers is employed to assess its safety during the whole sintering period.

#### 4.3.1. Competition Between Microwave Heating and Thermal Conductivity in Temperature

As the thermal stresses are significantly related to the temperature gradient, the factors influencing the temperature gradient were carefully analyzed.

**Figure 11:**
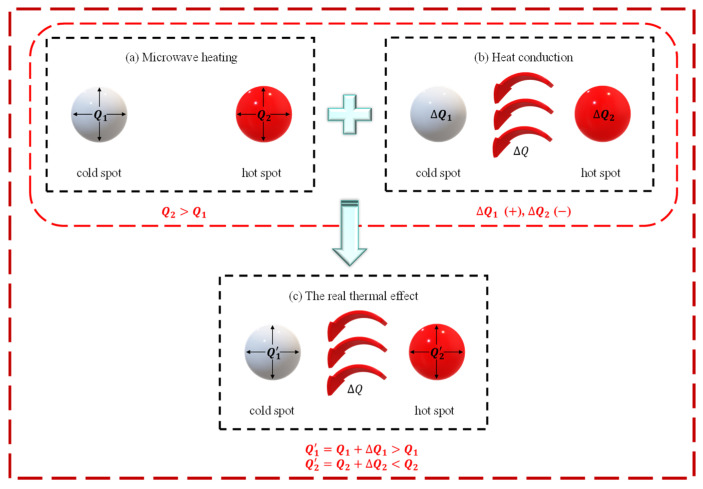
The modes of action of microwave sintering: (**a**) microwave heating, (**b**) heat conduction, and (**c**) real sintering process.

In regions around the cold spots with weak electric fields, the generated heat $Q_{1}$ increase is relatively small, while in areas around the hot spots with strong electric fields, the generated heat $Q_{2}$, and also the temperature increase, is relatively large, illustrated in Figure 11a. In other words, the microwave heating will intensify the temperature gradient. However, due to its poor thermal conductivity at the beginning and the significant attenuation of its conduction performance as the temperature rises, the heat accepted by the cold spot $∆Q_{1}$ or the heat sent by the hot spot $∆Q_{2}$, illustrated in Figure 11b is much less than $Q_{2}$. Resultantly, the net heat at hot spot $Q_{2}^{\prime }$ is much more than the net heat at cold spot $Q_{1}^{\prime }$, illustrated in Figure 11c, meaning the temperature gradient driven by the microwave heating could not be released by heat conductivity.

A quantitative analysis of the influence of the thermal conductivity on the temperature gradient was operated as follows, to clarify this competition. Interestingly, numerical simulations could easily obtain microwave heating by setting the thermal conductivity to 0, regardless of the effect of heat conduction on the temperature gradient.

For an accurate evaluation, it was critical for the thermal conductivity effect that the coefficient of thermal conductivity was considered temperature-dependent, as shown in Table 1. The nominal temperature gradient $\bar{\nabla T}$ of LiH after microwave heating for 10 s and 20 s is listed in Table 3 as group (a). For comparison, the real temperature field decided by the combined microwave heating and thermal conductivity is shown in Table 3 as group (b).

Subsequently, the mitigation effect on the temperature gradient driven by the thermal conduction could be obtained by the difference between group (a) and group (b). In detail, the value of 0.038 °C/mm is obtained by subtracting the result of case 1 from that of case 3, and the value of 0.132 °C/mm is acquired through subtracting the result of case 2 from that of case 4. However, both of these values are rather small when compared with those of case 1 and case 2, which involve pure heating. In group (a), and in the difference between group (a) and group (b), we found that the effect of microwave heating was significantly larger than that of thermal conductivity.

The nominal temperature gradient in case 3 was 0.158 °C/mm, which was not very large; however, when the sintering time was doubled in case 4, it increased to 0.261 °C/mm. This indicated that a longer heating time led to a larger temperature gradient. Several hours were required to increase the temperature to the target temperature (520 °C), and the accumulation of temperature gradients could cause fatal damage to the strength of the ceramic. This accumulation was actually the result of competition between microwave heating and thermal conductivity.

#### 4.3.2. Unit Cycle of Sintering–Resting Heating Method

According to the above quantitative analysis, a new heating strategy was proposed. To compensate for the effect of thermal conductivity, more time was provided for thermal conductivity, and a resting period was added after a sintering period to construct a unit sintering cycle. Therefore, the entire sintering process consisted of several such sintering cycles, termed the cyclic sintering–resting heating mode.

**Figure 12:**
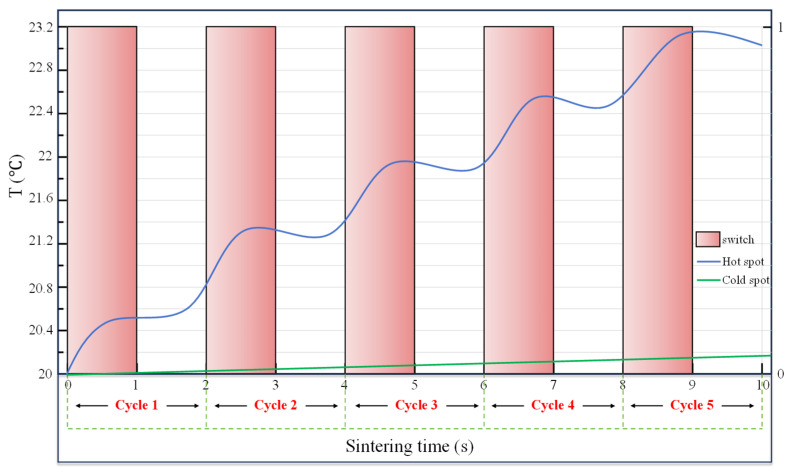
The temperature curve under the cyclic sintering–resting heating method.

A unit cycle of sintering–resting was characterized by the ratio of duty time of microwaves to the cycle. For example, we set the ratio to 0.5 and the cycle to 2 s, where the microwaves were fed in in the first second, but turned off in the next second, as shown in the abscissa from 0 to 2 in Figure 12. Figure 12 shows a sintering case with a cycle of 2 s repeated 5 times. The pink bar chart in Figure 12 demonstrates the microwave feed state, represented by the right axis, with 1 signifying on and 0 signifying off. The temperatures at the hot and cold spots are recorded by the blue curve and the green curve, respectively. It is represented by the left axis. The hot spot temperature increased rapidly when the microwave is on duty, named as sintering, but it rose slightly or even fell down in the resting stage, while the cold point temperature gently increased throughout the cycle, with the increase in the resting stage attributed to the thermal conductivity.

According to the method presented in Figure 12, a sintering–resting cycle with the ratio of duty time to cycle of 0.95 and a cycle of 210 was applied to the LiH sample. After the 200 s sintering stage, the hot spot temperature reached 83.5442 °C, and the cold spot temperature reached 65.206 °C. The corresponding nominal temperature gradient was 0.489 °C/mm. We further confirmed that the maximum thermal stress at this moment was 13.819 MPa. After a subsequent 10 s of resting, the hot spot temperature decreased to 79.835 °C, and the cold spot temperature increased to 67.285 °C. The corresponding nominal temperature gradient decreased to 0.335 °C/mm, and the maximum thermal stress at this moment obviously decreased by about 36%, to 8.8024 MPa. It is reasonable to believe that a cyclic sintering–resting heating method is useful in sintering such materials as LiH ceramics.

#### 4.3.3. Parameter Design of the Sintering–Resting Heating Mode for Sintering LiH Based on PFAD

Considering strength requirements and sintering efficiency, the resting stage was thoroughly evaluated, which was sufficient to balance the increase in temperature gradient without a loss in efficiency. Therefore, flexible, but not robotic, unit cycles of sintering–resting mode were recommended until the temperature achieved the target sintering temperature.

Note the tensile strength of LiH shown in Table 1, which does not perform badly below 300 °C, but really poorly above 300 °C; 300 °C is selected to be a dividing line in the parameter design of the sintering–resting heating mode, defining the rapid heating stage and the precise temperature control stage.

**Figure 13:**
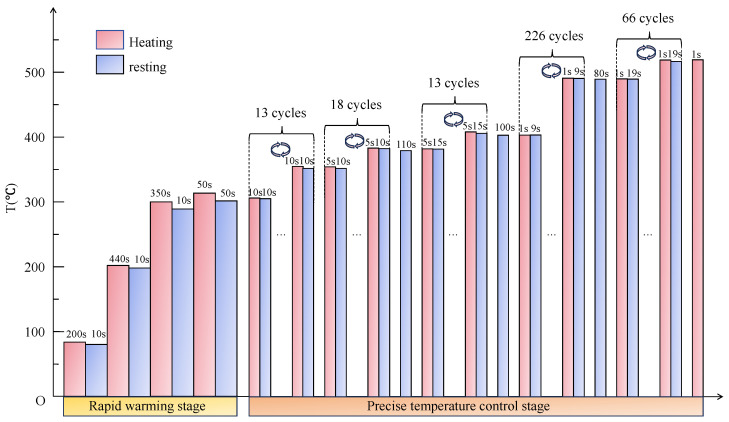
Design for the microwave sintering of LiH ceramics.

**Figure 14:**
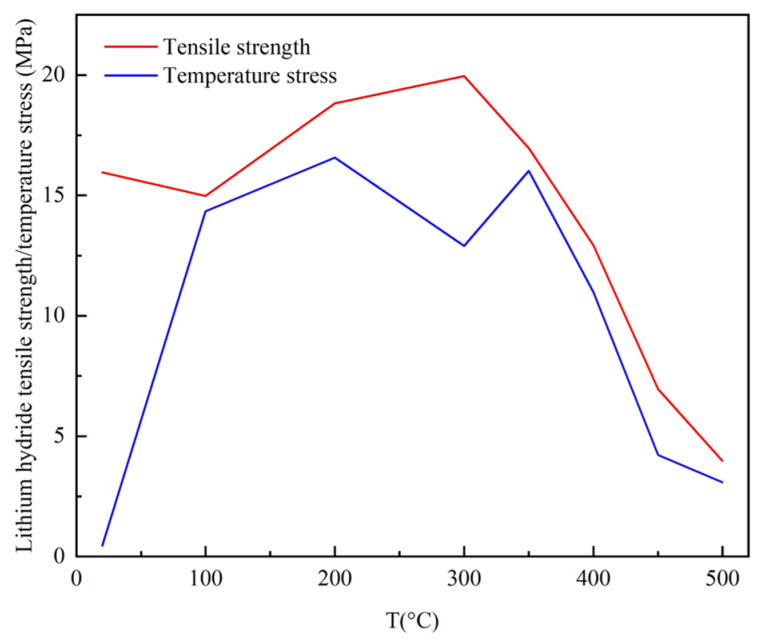
Safety assessment diagram of the entire microwave sintering process of LiH ceramics.

As shown in the process-based failure assessment diagram (PFAD), the perfect LiH ceramic could be made when the thermal stress was consistently below the strength envelope. Accordingly, the thermal stresses were continuously calculated. Once the stress exceeded or was close to the corresponding strength, the relevant sintering–resting unit cycles required to be modified. As a result, for the specific LiH ceramic sample, the rapid heating stage is subdivided into 4 cycles; however, the precise temperature control stage was subdivided more finely into 336 cycles, with details shown in Figure 13. Figure 14 presents the corresponding maximum thermal stresses, which were below the strength envelope up to 520 °C, demonstrating safe sintering at the designed working parameters.

In summary, 3349 s is adopted as the resting time and 2522 s as the sintering time: in total, 5871 s, about 1.63 h, is the cost of heating the LiH sample to the target sintering temperature (520 °C), indicating amazing efficiency compared with the former experience of the researchers.

Notably, the results presented here only relate to the specific dimensions of LiH ceramics and microwave cavities. Utilizing the cyclic sintering–resting method, the parameters could also be obtained if other dimensions of LiH ceramic and microwave cavities are required. While the current parameters conservatively ensure safety, future work could optimize the sintering–resting ratio using machine learning techniques, provided real-time stress monitoring becomes available in industrial furnaces.

## 5. Conclusions

LiH has gained increasing importance as a material for hydrogen production, hydrogen storage, and neutron shielding.

In this study, we first adjust the heating position and rotate the sample, but find that it is not sufficient to effectively control the uniformity of its temperature rise, due to the material’s inferior properties. Next, we quantify the competition between microwave heating and thermal conductivity in temperature. The results show that the microwave heating effect is approximately 3 to 5 times that of the heat conduction effect and is the main factor hindering the safe sintering of LiH ceramics to the target temperature. Finally, according to the imbalance between microwave heating and heat conduction, we propose a novel microwave cyclic sintering–resting strategy and design a precise set of working parameters for a specific LiH ceramic sample, in which the sintering time is substantially reduced from several days to about 1.63 h, effectively sintering LiH to the target temperature.

The theoretical and technical achievements of this work have contributed to the project initiated by this funding in 2020 and laid an important foundation for the true attempt to apply microwave sintering technology in the industrial production of lithium hydride ceramics.

## Figures and Tables

**Table 1:** Thermodynamic and physical properties of LiH [4,5,25,29].

Temperature (°C)	Specific Heat (J/kg/°C)	Density (kg/m^3^)	Thermal Conductivity (W/m/°C)	Expansion Coefficient (10^−6^/°C)	Young’s Modulus (GPa)	Poisson’s Ratio	Tensile Strength (MPa)
20	3716.25	770	6.904	26.8	47.3	0.08	15.96
100			6.527	35.5	44.7	0.10	14.98
150			/	39.4	/	/	/
200			5.732	41.3	42.3	0.11	18.82
250			/	42.4	/	0.11	/
300			5.146	43.4	40.2	0.11	19.96
350			/	44.2	/	/	16.97
400			4.686	46.0	38.0	0.12	12.93
450			/	47.9	/	/	6.95
500			4.310	49.5	36.4	0.12	3.98
600			4.100	/	35.5	0.11	0.77

**Table 2:** Distribution of hot and cold spots after microwave heating for 10 s.

Dielectric Loss Factor	Distance (mm)	Cold Point Temperature (°C)	Hot Point Temperature (°C)	$\bar{\nabla T}$ (°C/mm)	COV
0.01	37.498	20.445	26.055	0.149	0.35
0.02		20.58	27.892	0.195	0.37
0.03		20.713	29.7	0.240	0.41
0.04		20.845	31.478	0.284	0.43

**Table 3:** Comparison of nominal temperature gradient under thermal conductivity and heating.

Group	Case	Heating Time (s)	Thermal Conductivity	Temperature at Cold Spots (°C)	Temperature at Hot Spots (°C)	Nominal Temperature Gradient (°C/mm)
(a)	1	10	0	20.001	27.367	0.196
	2	20	0	20.002	34.735	0.393
(b)	3	10	K(T)	20.449	26.39	0.158
	4	20	K(T)	21.558	31.356	0.261

## Data Availability

The original contributions presented in this study are included in the article. Further inquiries can be directed to the corresponding author.
